# Reevaluation and Classification of Duodenal Lesions in B6C3F1 Mice and F344 Rats from 4 Studies of Hexavalent Chromium in Drinking Water

**DOI:** 10.1177/0192623315611501

**Published:** 2015-11-04

**Authors:** John M. Cullen, Jerrold M. Ward, Chad M. Thompson

**Affiliations:** 1 College of Veterinary Medicine, North Carolina State University, Raleigh, North Carolina, USA; 2 Global VetPathology, Montgomery Village, Maryland, USA; 3 ToxStrategies Inc., Katy, Texas, USA

**Keywords:** mouse pathology, rat pathology, gastrointestinal system, environmental toxicology, cell proliferation, carcinogenesis

## Abstract

Thirteen-week and 2-year drinking water studies conducted by the National Toxicology Program (NTP) reported that hexavalent chromium (Cr(VI)) induced diffuse epithelial hyperplasia in the duodenum of B6C3F1 mice but not F344 rats. In the 2-year study, Cr(VI) exposure was additionally associated with duodenal adenomas and carcinomas in mice only. Subsequent 13-week Cr(VI) studies conducted by another group demonstrated non-neoplastic duodenal lesions in B6C3F1 mice similar to those of the NTP study as well as mild duodenal hyperplasia in F344 rats. Because intestinal lesions in mice are the basis for proposed safety standards for Cr(VI), and the histopathology data are relevant to the mode of action, consistency (an important Hill criterion for causality) was assessed across the aforementioned studies. Two veterinary pathologists applied uniform diagnostic criteria to the duodenal lesions in rats and mice from the 4 repeated-dose studies. Comparable non-neoplastic intestinal lesions were evident in mice and rats from all 4 studies; however, the incidence and severity of intestinal lesions were greater in mice than rats. These findings demonstrate consistency across studies and species and highlight the importance of standardized nomenclature for intestinal pathology. The differences in the severity of non-neoplastic lesions also likely contribute to the differential tumor response.

## Introduction

In 2007 and 2008, the National Toxicology Program (NTP) released reports that described the toxic and carcinogenic effects of hexavalent chromium (Cr(VI)) in 13-week and 2-year rodent drinking water studies (NTP 2007, 2008b). Toxicity and carcinogenicity studies involving trivalent chromium (Cr(III)) were released at the same time (NTP 2008a). Results from the 2-year studies of Cr(VI) and Cr(III) were subsequently published in the peer-reviewed literature (Stout, Herbert, et al. 2009; Stout, Nyska, et al. 2009). Cr(VI) exposure was associated with oral tumors in F344 rats and adenomas and carcinomas of the duodenum and jejunum in B6C3F1 mice (Stout, Herbert, et al. 2009). Despite higher milligram per kilogram body weight doses, Cr(III) exposure was not associated with increased tumors in any organ (Stout, Nyska, et al. 2009). These disparate findings are consistent with the lower bioavailability of Cr(III) relative to Cr(VI) (De Flora et al. 1997).

The major non-neoplastic lesions reported in the duodenum of B6C3F1 mice exposed to Cr(VI) were diffuse epithelial hyperplasia, histiocytic infiltration in the lamina propria of the villus mucosa, blunted villi, and generalized mucosal epithelial hypercellularity (Stout, Herbert, et al. 2009). In both the 13-week and 2-year studies, these effects were characterized as having occurred secondary to mucosal injury (NTP 2007, 2008b). In contrast to mice, histiocytic infiltration was the only duodenal effect reported in F344 rats. According to NTP (2008b), rats did not exhibit mucosal injury or hyperplasia and did not develop intestinal tumors. Considering that comparable drinking water concentrations were administered to both species, the different outcomes suggested species differences in pharmacokinetic and/or pharmacodynamic responses to Cr(VI).

Between 2011 and 2012, two 13-week studies were conducted to investigate the mode of action (MOA) of Cr(VI) in the rodent small intestine (Thompson et al. 2011; Thompson, Proctor, et al. 2012). Intestinal lesions in the duodenum of B6C3F1 mice as reported by Thompson et al. (2011) were similar to the lesions observed in B6C3F1 mice in NTP (2007), whereas intestinal lesions observed in the duodenum of F344 rats differed from those observed in F344 rats in the NTP (2007) study. In fact, the lesions observed in rats were similar, albeit milder, to those observed in B6C3F1 mice (Thompson, Proctor, et al. 2012). The differences in the two 13-week rat studies were posited to relate to disparities in water/Cr(VI) intake between the 2 studies (Thompson, Proctor, et al. 2012). And although the highest Cr(VI) concentration differed in the 2 studies, the estimated milligram per kilogram doses were comparable. Therefore, other factors likely contributed to the apparent absence of intestinal lesions in F344 rats in the NTP (2007) study as well as the chronic 2-year bioassay (NTP 2008b).

Subsequent studies indicated that toxicogenomic responses were similar in the duodenum of rats and mice from the 13-week studies conducted by Thompson et al. (Kopec, Kim, et al. 2012; Kopec, Thompson, et al. 2012). Synchrotron-based X-ray fluorescence microscopy revealed the presence of chromium in duodenal villi (but not crypts) of both species (Thompson, Seiter, et al. 2015). The interspecies similarities in histopathologic results, gene expression, and chromium localization in these studies suggested that subtle histological findings might have been overlooked in F344 rats of the NTP study. If so, the present lack of consistency across studies of F344 rats might be inaccurate; moreover, the lack of consistency in non-neoplastic intestinal lesions reported in rats and mice of the NTP studies might also be misleading. Consistency is an important element of the Hill criteria for establishing causality (U.S. Environmental Protection Agency [U.S. EPA] 2005). Consistency is strengthened when similar effects are observed in multiple studies, and when a similar sequela is observed in multiple species.

Therefore, we obtained permission from the NTP to reexamine selected non-neoplastic duodenal lesions in rats and mice from their 13-week and 2-year Cr(VI) drinking water studies (NTP 2007, 2008b). A limited retrospective peer review was conducted with the primary objective to review the duodenal lesions and determine if there were any differences in duodenal lesions in mice and rats and any differences between the NTP and Thompson et al. Cr(VI) studies. Herein, we used diagnostic criteria and terminology that best characterized the non-neoplastic effects of Cr(VI) in the small intestine of both species and subsequently applied these criteria consistently during a review of selected histopathological sections from the NTP 13-week and 2-year studies in mice and rats as well as the 13-week studies by Thompson et al. (2011) and Thompson, Proctor, et al. (2012).

## Methods

Histologic sections of duodenum were evaluated jointly by 2 American College of Veterinary Pathologists (ACVP) board-certified veterinary pathologists (J.M.C. and J.M.W.). The specific goals of this review were (1) to develop a duodenal lesion classification in mice and rats; (2) to determine the degree to which non-neoplastic duodenal findings in the NTP and Thompson et al. studies were qualitatively different or similar; (3) to determine the degree to which findings in rats were qualitatively different or similar to those observed in mice; and (4) to determine whether the various lesions are likely representative of a single pathologic process versus independent responses. A summary of the different studies and numbers of slides reviewed from each study is shown in Table 1. Slides for examination were selected impartially and consecutively. Exceptions included slides in which there was substantial autolytic change in the sections, or missing slides, in which cases the next consecutive slides were selected. A single section of duodenum was available and examined per animal.

**Table 1.** Duodenal Sections Reviewed.

Study	Species	Exposure duration	Sex	Dose groups evaluated (sodium dichromate dihydrate, milligram per liter)	Total number of animals examined
NTP (2007, 2008)	B6C3F1 mice	13 Weeks	M	0, 1,000	20
		13 Weeks	F	0, 1,000	19
		2 Years	M	0, 14.3, 28.6, 85.7, 257.4	51
		2 Years	F	0, 14.3, 57.3, 172, 516	50
	F344 rats	13 Weeks	M	0, 500, 1,000	30
		13 Weeks	F	0, 1,000	20
		2 Years	M	0, 57.3, 172, 516	43
		2 Years	F	0, 172, 516	40
Thompson et al. (2011)	B6C3F1 mice	13 Weeks	F	0, 60, 170, 520	40
Thompson et al. (2012)	F344 rats	13 Weeks	F	0, 170, 520	30
				Total	343

A qualitative evaluation of slides from the NTP and Thompson et al. studies was done to establish the most appropriate diagnostic criteria and terminology to be used for the review. The final lexicon (see third section) was derived from the experience of the pathologists, current convention, and consultation of the scientific literature (Ward and Treuting 2014; Bertram, Markovits, and Julian 1996). The selected slides were reviewed via brightfield microscopy without knowledge of the prior individual animal diagnoses. Findings were scored for severity according to the following system: grade 0 = not remarkable, grade 1 = minimal, grade 2 = mild, and grade 3 = moderate. No severe changes were observed in any of the examined slides. The duodenal sections reviewed usually did not contain Brunner’s glands which occur in the most proximal portion of the duodenum.

In the NTP and Thompson et al. studies, rodents were exposed to Cr(VI) in the form of sodium dichromate dihydrate (SDD). Nominally, the estimated Cr(VI) concentration was ∼34.9% of the SDD dose (e.g., 516 mg/L SDD is equivalent to 180 mg/L Cr(VI)). All studies were conducted at Southern Research (Birmingham, AL), thus minimizing interlaboratory variability. In all studies, rodents were provided *ad libitum* access to irradiated NTP-2000 Wafers (Zeigler Bros., Gardners, PA) and water or test article. Conditions in the 13-week studies by Thompson et al. matched the 13-week NTP studies to the extent possible. However, B6C3F1 mice and F344/N rats in the NTP 13-week studies were obtained from Taconic Farms (Germantown, NY), and studies took place between November 2001 and February 2002. In contrast, B6C3F1/Crl mice were obtained from Charles River (Raleigh, NC) and placed on test from March 2010 to June 2010 in Thompson et al. (2011). F344/IcoCrl rats were obtained from Charles River (Stone Ridge, NY) and placed on test from August 2010 and November 2010 in Thompson, Proctor, et al. (2012). The Thompson et al. studies only included female rodents because the studies were designed to investigate MOA as opposed to hazard identification, and the NTP studies previously reported similar responses in both sexes. The average amount of water (or test article) consumed per day differed between the studies. For example, average consumption in week 1 and week 13 in control female mice was, respectively, 4.9 and 6.8 ml/day in Thompson et al. (2011) and 3.0 and 3.3 ml/day in NTP (2007). In control female rats, the average week 1 and week 13 consumption was 23.6 and 20.7 ml/day in Thompson, Proctor, et al. (2012) and 14.1 and 11.2 ml/day in NTP (2007). These differences in consumption led to different milligram per kilogram body weight intakes at comparable drinking water concentrations and, in addition to potential colony differences, likely contributed to interstudy variability in response to Cr(VI).

## Results

### Diagnostic Criteria

Following an initial duodenal slide review, it was determined that potential treatment-related non-neoplastic findings in the duodenum of mice and rats were limited to the following 5 histopathologic diagnoses: (1) villus, histiocytic cellular infiltrates; (2) villus, atrophy/blunting; (3) villus, enterocyte vacuolation; (4) villus, single-cell necrosis; and (5) crypt, epithelial hyperplasia. With rare exceptions, such findings were not present in control animals, and the prevalence and/or severity of these findings often increased in a dose-responsive manner in SDD-treated animals.

Histiocytic infiltration occurred primarily in the lamina propria of the villus tips and was characterized by small nodular aggregations of histiocytic macrophages with abundant, faintly granular, and eosinophilic cytoplasm. These macrophages occasionally formed multinucleated syncytia. Villi were considered atrophied/blunted when they appeared shortened and/or thickened relative to those of the control animals. Enterocyte vacuolation presented as single or multiple, sharply defined, clear, or slightly flocculent spaces within the cytoplasm of terminal villus enterocytes. Single-cell necrosis in the villous tips appeared either as cells with condensed cytoplasm and smudged nuclei with a peripheral halo (apoptotic appearance) or more often as cells with karyorrhectic nuclei that were located either within the villus epithelium or within the lamina propria. It could not always be readily determined whether such cells were originally enterocytes or inflammatory cells. Crypt epithelial hyperplasia was characterized by elongated crypts that were lined by increased numbers of crowded tall enterocytes with hyperchromatic basophilic cytoplasm and nuclear chromatin clumping. In more extensively affected cases (mice especially), the hyperplastic enterocytes of villi additionally displayed increased cell height and tinctorial changes compared to those of controls.

### Incidence and Severity of Duodenal Lesions in 13-Week Studies

**Figure 1.**
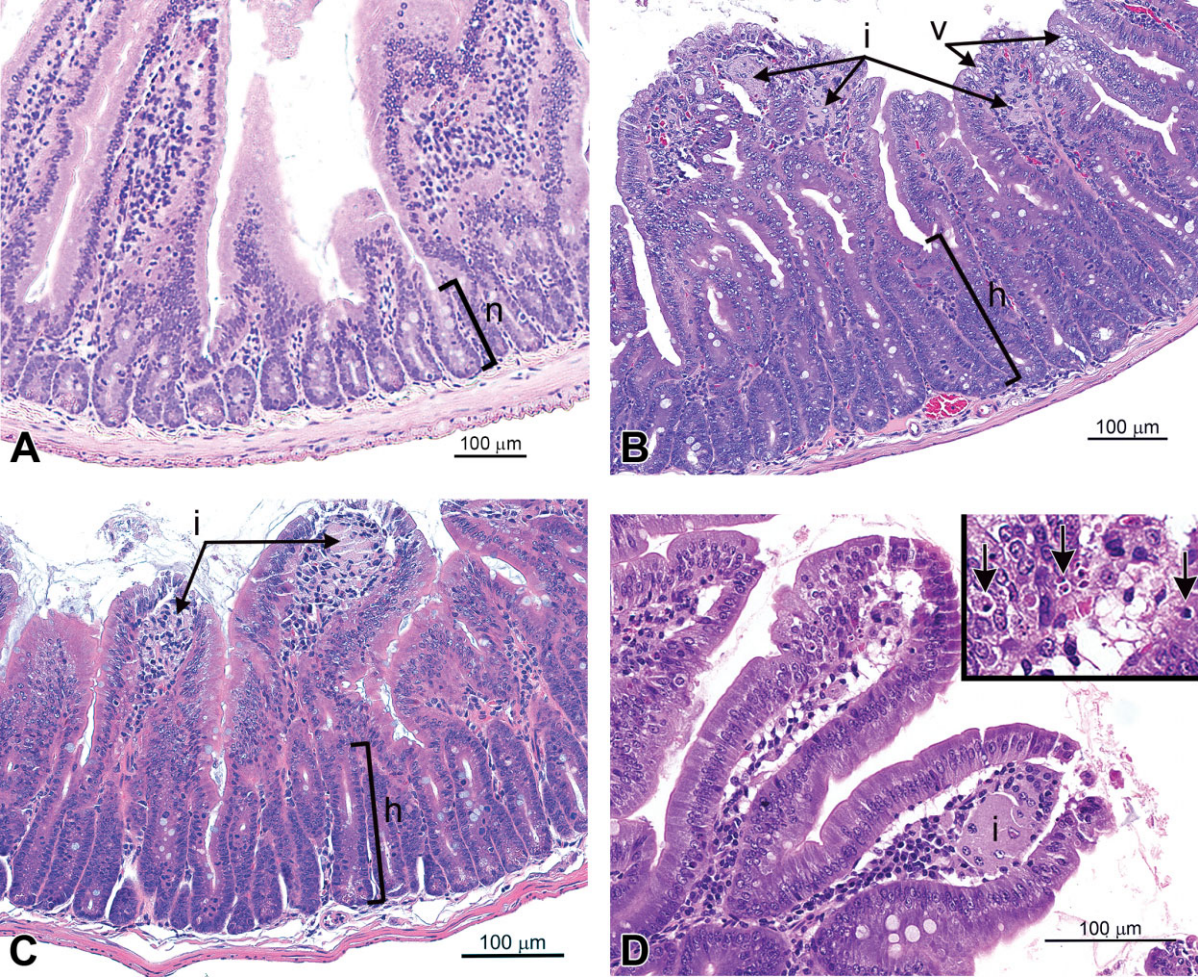
Histopathologic findings in mice. (A) Duodenum of a control mouse illustrating the appearance of the normal crypt epithelium (*n*) in an untreated animal (Thompson et al. 2011). (B) Duodenal findings in this sodium dichromate dihydrate (SDD)-treated mouse included moderate histiocytic infiltrates (i), moderate villus atrophy and blunting, moderate villus enterocyte vacuolation (v), minimal single-cell necrosis in villi, and moderate crypt epithelial hyperplasia (h). Female mouse exposed to 520 mg/L SDD for 13 weeks from Thompson et al. (2011). (C) Duodenal villus atrophy and blunting in this NTP mouse are comparable to changes described in the duodenum in (B), except that crypt epithelial hyperplasia (h) was slightly less florid, and enterocyte vacuolation was not apparent, (i) histiocytic infiltrates. Female mouse exposed to 1,000 mg/L SDD for 13 weeks from NTP (2007). (D) Duodenal single-cell necrosis of the villi (arrows) is apparent in this high-magnification view and inset. Such necrosis was not observed in duodena of control mice. Histiocytic infiltrates (i) occasionally formed syncytia, as in this case. Male mouse exposed to 257.4 mg/L SDD for 2 years from NTP (2008b). All staining is H&E.

**Figure 2.**
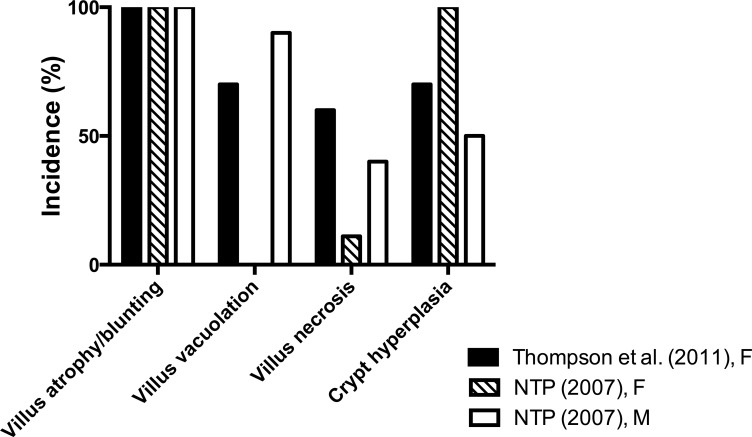
Incidence of duodenal lesions in mice from 13-week studies found in a reevaluation of the slides. F, female and M, male.

The incidence and severity of duodenal lesions in selected dose groups of female mice from Thompson et al. (2011) and male and female mice from NTP (2007) were reevaluated (by J.M.C. and J.M.W.), and the results are summarized in Table 2 and illustrated in Figure 1A–C. In the highest dose groups of each study (∼80–90 mg/kg SDD), there was 100% incidence of histiocytic infiltration and atrophy/blunting of duodenal villi. Female mice from Thompson et al. (2011) and male mice from NTP (2007) exhibited high incidences of villus enterocyte vacuolation; however, this effect was not present in female mice from NTP (2007). Single-cell necrosis (possibly apoptosis) was evident in 6/10 female mice from Thompson et al. (2011), 4/10 male mice from NTP (2007), and only 1/9 female mice from NTP (2007). In the crypt compartment, epithelial hyperplasia was present in 5/10 male mice (NTP 2007), 9/9 female mice (NTP 2007), and 7/10 female mice from Thompson et al. (2011). With the exception of villus enterocyte vacuolation and single-cell necrosis, the incidences of lesions at comparable milligram per kilogram SDD doses were similar in male and female mice from the NTP (2007) and Thompson et al. (2011) studies (Figure 2).

**Table 2.** Reevaluation of Duodenal Lesions in B6C3F1 Mice in the 13-Week Drinking Water Studies.

Study		Thompson et al. (2011), female				NTP (2007), female		NTP (2007), male	
Nominal concentration milligram per liter SDD		0	60	170	520	0	1,000	0	1,000
Nominal dose milligram per kilogram SDD		0	13	33	89	0	80	0	80
Number of mice examined		10	10	10	10	10	9	10	10
Villus histiocytic cellular infiltrates	Grade 1	0^a^	1	2	2	0	3	0	3
	Grade 2	0	0	8	4	0	6	0	7
	Grade 3	0	0	0	4	0	0	0	0
	All grades	0	1	10	10	0	9	0	10
Villus atrophy/blunting	Grade 1	0	4	8	6	0	5	0	1
	Grade 2	0	0	2	1	0	4	0	7
	Grade 3	0	0	0	3	0	0	0	2
	All grades	0	4	10	10	0	9	0	10
Villus enterocyte vacuolation	Grade 1	0	2	6	2	0	0	0	6
	Grade 2	0	1	2	2	0	0	0	3
	Grade 3	0	0	0	3	0	0	0	0
	All grades	0	3	8	7	0	0	0	9
Villus single-cell necrosis	Grade 1	0	0	0	6	0	1	0	4
	Grade 2	0	0	0	0	0	0	0	0
	Grade 3	0	0	0	0	0	0	0	0
	All grades	0	0	0	6	0	1	0	4
Crypt epithelial hyperplasia	Grade 1	0	3	6	2	0	9	0	4
	Grade 2	0	0	0	3	0	0	0	1
	Grade 3	0	0	0	2	0	0	0	0
	All grades	0	3	6	7	0	9	0	5

*Note:* SSD = sodium dichromate dihydrate.

^a^Numbers of animals with lesions.

**Figure 3.**
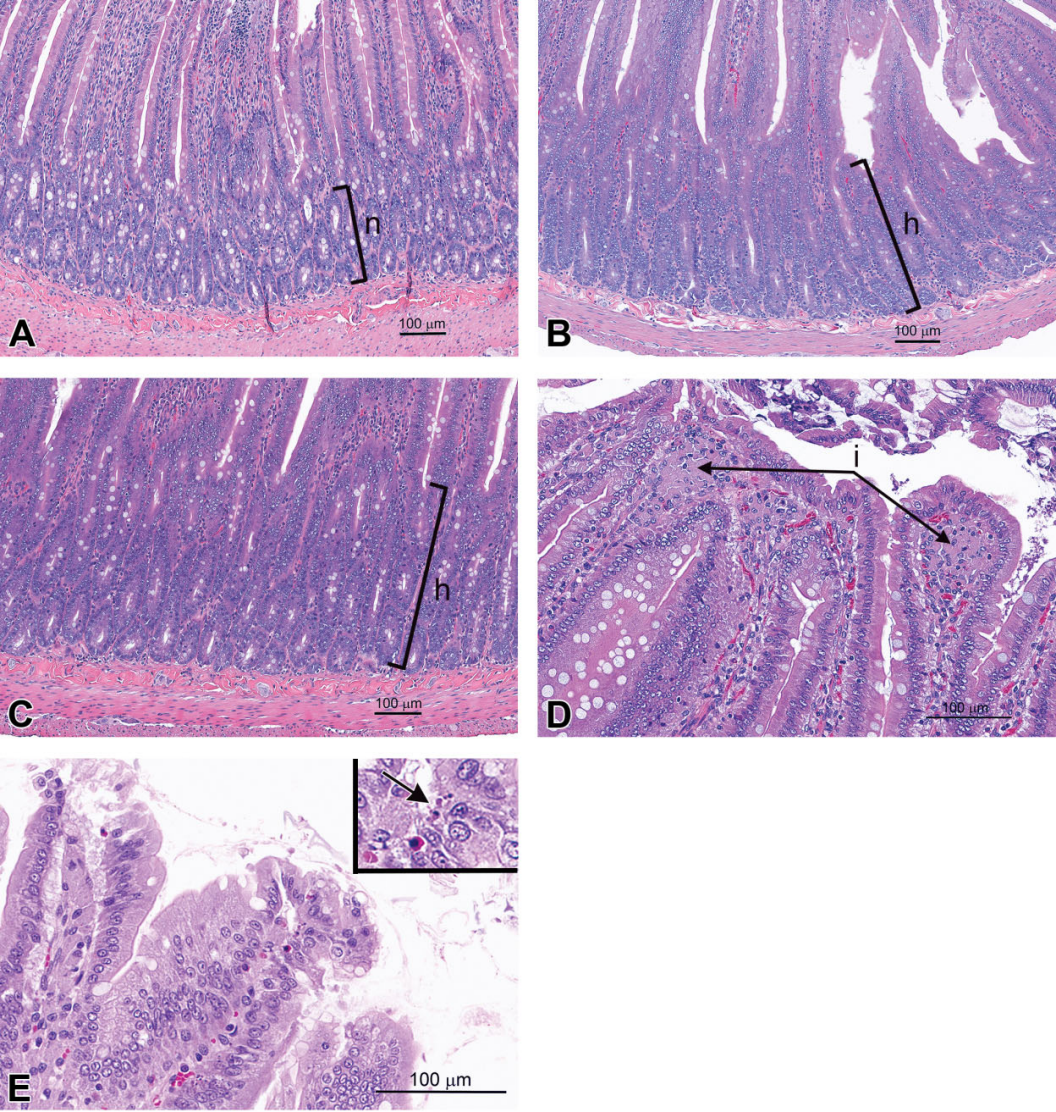
Histopathologic findings in rats. (A) Duodenum of a control male rat illustrating the thickness and appearance of the normal crypt epithelium (*n*) in an untreated animal (NTP, 2007). (B) In this SDD-treated rat, the duodenal crypt epithelium is hyperplastic (thickening of the proliferative crypt zone, h), and crypt and villus epithelial cells display a high degree of morphologic similarity (compare to the normal rat duodenum in 3A). Female rat exposed to 520 mg/L SDD for 13 weeks from Thompson et al. (2012). (C) The appearance of the hyperplastic duodenal crypt epithelium (h) of this NTP study rat is very similar to that seen in (B). Female rat exposed to 1,000 mg/L SDD for 13 weeks from NTP (2007). (D) The duodenal villus tips are blunted and histiocytic infiltrates (i) are again evident. Male rat exposed to 1,000 mg/L SDD for 3 weeks from NTP (2007). (E) Minimal duodenal single-cell necrosis (arrow, inset) is evident in a slightly blunted villus tip in this image. Male rat exposed to 516 mg/L SDD for 2 years from NTP (2008b). All staining is H&E.

The incidence and severity of duodenal lesions in select dose groups in female rats from Thompson, Proctor, et al. (2012) and male and female rats from NTP (2007) were reevaluated (by J.M.C. and J.M.W.) and the results are summarized in Table 3 and illustrated in Figure 3A–D. In the highest dose groups of each study (∼60 mg/kg SDD), the incidence of villus histiocytic infiltration was 90–100%. Villus atrophy/blunting was evident in 8/10 female rats from Thompson, Proctor, et al. (2012) but not in rats from NTP (2007). Villus enterocyte vacuolation was not evident in any rats from either study. One of 10 male rats (NTP 2007) and 3/10 female rats (Thompson, Proctor, et al. 2012) exhibited villus single-cell necrosis. Crypt epithelial hyperplasia was evident in 7/10 male rats (NTP 2007) and only 3/10 female rats from NTP (2007) and 2/10 female rats from Thompson, Proctor, et al. (2012). Crypt epithelial hyperplasia was also observed in male rats exposed to 32 mg/kg SDD (5/10; NTP 2007), and female rats exposed to 20 mg/kg SDD (3/10; Thompson, Proctor, et al. 2012). Overall, the prevalence and severity of histiocytic cellular infiltrates and crypt epithelial hyperplasia were comparable between the NTP (2007) and Thompson, Proctor, et al. (2012) studies. Atrophy/blunting and single-cell necrosis were not observed in NTP female rats.

**Table 3.** Reevaluation of Duodenal Lesions in F344 Rats in the 13-week Drinking Water Studies.

Study		Thompson et al. (2012), female			NTP (2007), female		NTP (2007), male		
Nominal concentration milligram per liter SDD		0	170	520	0	1,000	0	500	1,000
Nominal dose milligram per kilogram SDD		0	20	59	0	61	0	32	60
Number of rats examined		10	10	10	10	10	10	10	10
Villus histiocytic cellular infiltrates	Grade 1	0^a^	9	5	1	7	0	4	6
	Grade 2	0	1	4	0	2	0	1	4
	Grade 3	0	0	0	0	0	0	0	0
	All grades	0	10	9	1	9	0	5	10
Villus atrophy/blunting	Grade 1	0	4	8	0	0	0	0	0
	Grade 2	0	0	0	0	0	0	0	0
	Grade 3	0	0	0	0	0	0	0	0
	All grades	0	4	8	0	0	0	0	0
Villus enterocyte vacuolation	Grade 1	0	0	0	0	0	0	0	0
	Grade 2	0	0	0	0	0	0	0	0
	Grade 3	0	0	0	0	0	0	0	0
	All grades	0	0	0	0	0	0	0	0
Villus single-cell necrosis	Grade 1	0	0	3	0	0	0	0	1
	Grade 2	0	0	0	0	0	0	0	0
	Grade 3	0	0	0	0	0	0	0	0
	All grades	0	0	3	0	0	0	0	1
Crypt epithelial hyperplasia	Grade 1	0	3	2	0	3	0	5	6
	Grade 2	0	0	0	0	0	0	0	1
	Grade 3	0	0	0	0	0	0	0	0
	All grades	0	3	2	0	3	0	5	7

*Note:* SSD = sodium dichromate dihydrate.

^a^Numbers of animals with lesions.

### Incidence and Severity of Non-neoplastic Duodenal Lesions in NTP 2-Year Cancer Bioassay

**Figure 4.**
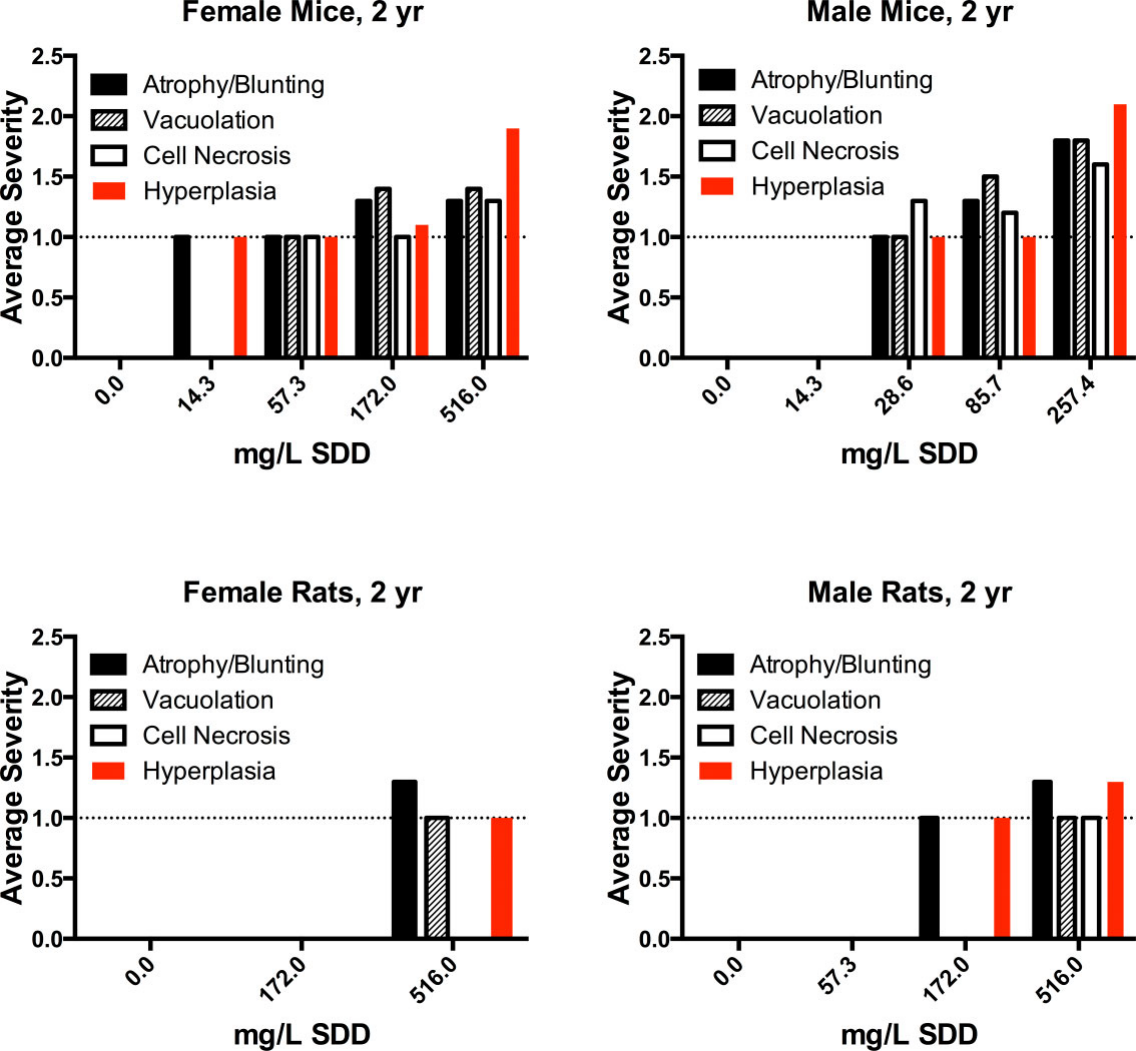
Average severity scores for histopathological lesions in the duodenum of rats and mice in the NTP 2-year cancer bioassay found in a reevaluation of the slides. Only 10–15 animals/slides were reviewed out of a possible 50 animals/slides per treatment group. Bars represent weighted averages among animals with each specific lesion (unaffected animals are not included). Atrophy/blunting, vacuolation, and necrosis refer to the effects in villi, whereas hyperplasia refers to crypt epithelial cell hyperplasia. *Note:* Plot does not include the one control female rat with grade 3 atrophy/blunting in Table 5.

The incidence and severity of non-neoplastic duodenal lesions in select mice from all dose groups of the 2-year cancer bioassay (NTP 2008b) were reevaluated (by J.M.C. and J.M.W.), and the results are summarized in Table 4 and illustrated in Figures 1D and 4. In the highest dose groups for each sex (∼17 to 25 mg/kg SDD), there were comparable incidences and severities for all 5 types of intestinal findings. A similar outcome was observed in the second highest dose groups (∼7 to 9 mg/kg SDD), although the incidence of atrophy/blunting was greater in male mice. Overall, the effects were similar in male and female mice at comparable milligram per kilogram dose levels.

**Table 4.** Reevaluation of Non-neoplastic Duodenal Lesions in B6C3F1 Mice in the NTP 2-year Drinking Water Studies.

Sex		Female					Male				
Nominal concentration milligram per liter SDD		0	14.3	57.3	172	516	0	14.3	28.6	85.7	257.4
Nominal dose milligram per kilogram SDD		0	1.1	3.9	9	25	0	1.1	2.6	7	17
Number of mice examined		9	10	10	10	11	10	10	11	10	10
Villus histiocytic cellular infiltrates	Grade 1	0	0^a^	0	8	1	0	1	3	2	0
	Grade 2	0	0	0	1	9	0	0	0	7	8
	Grade 3	0	0	0	0	0	0	0	0	0	1
	All grades	0	0	0	9	10	0	1	3	9	9
Villus atrophy/blunting	Grade 1	0	2	1	2	7	0	0	2	6	4
	Grade 2	0	0	0	1	3	0	0	0	2	2
	Grade 3	0	0	0	0	0	0	0	0	0	2
	All grades	0	2	1	3	10	0	0	2	8	8
Villus enterocyte vacuolation	Grade 1	0	0	1	3	4	0	0	2	1	1
	Grade 2	0	0	0	2	3	0	0	0	1	5
	Grade 3	0	0	0	0	0	0	0	0	0	0
	All grades	0	0	1	5	7	0	0	2	2	6
Villus single-cell necrosis	Grade 1	0	0	2	3	5	0	0	2	4	2
	Grade 2	0	0	0	0	2	0	0	1	1	3
	Grade 3	0	0	0	0	0	0	0	0	0	0
	All grades	0	0	2	3	7	0	0	3	5	5
Crypt epithelial hyperplasia	Grade 1	0	2	4	7	2	0	0	3	8	1
	Grade 2	0	0	0	1	7	0	0	0	0	6
	Grade 3	0	0	0	0	1	0	0	0	0	2
	All grades	0	2	4	8	10	0	0	3	8	9

*Note:* SSD = sodium dichromate dihydrate.

^a^Numbers of animals with lesions.

The incidence and severity of duodenal lesions in select dose groups of rats from the 2-year cancer bioassay (NTP 2008b) were reevaluated (by J.M.C. and J.M.W.), and the results are summarized in Table 5 and illustrated in Figures 3E and 4. In the highest dose for each sex (∼17 to 20 mg/kg SDD), the effects were more prevalent in male rats than female rats. Villus atrophy/blunting was present in 7/11 male rats and 3/15 female rats. Villus enterocyte vacuolation was uncommon (1/11 males and 2/15 females). Single-cell necrosis was present in only 1 male rat. Crypt epithelial hyperplasia was present in only 2/15 female rats but was present in 8/11 male rats. Overall, the effects in female rats were minimal compared to those in male rats.

**Table 5.** Reevaluation of Non-neoplastic Duodenal Lesions in F344 Rats in the NTP 2-year Drinking Water Studies.

Sex		Female			Male			
Nominal concentration milligram per liter SDD		0	172	516	0	57.3	172	516
Nominal dose milligram per kilogram SDD		0	7	20	0	2.2	6	17
Number of rats examined		15	10	15	11	11	10	11
Villus histiocytic cellular infiltrates	Grade 1	0^a^	6	8	0	1	10	3
	Grade 2	0	0	4	0	0	0	7
	Grade 3	0	0	0	0	0	0	1
	All grades	0	6	12	0	1	10	11
Villus atrophy/blunting	Grade 1	0	0	2	0	0	2	5
	Grade 2	0	0	1	0	0	0	2
	Grade 3	1	0	0	0	0	0	0
	All grades	1	0	3	0	0	2	7
Villus enterocyte vacuolation	Grade 1	0	0	2	0	0	0	1
	Grade 2	0	0	0	0	0	0	0
	Grade 3	0	0	0	0	0	0	0
	All grades	0	0	2	0	0	0	1
Villus single-cell necrosis	Grade 1	0	0	0	0	0	0	1
	Grade 2	0	0	0	0	0	0	0
	Grade 3	0	0	0	0	0	0	0
	All grades	0	0	0	0	0	0	1
Crypt epithelial hyperplasia	Grade 1	0	0	2	0	0	5	6
	Grade 2	0	0	0	0	0	0	2
	Grade 3	0	0	0	0	0	0	0
	All grades	0	0	2	0	0	5	8

*Note:* SSD = sodium dichromate dihydrate.

^a^Numbers of animals with lesions.

## Discussion

In the 2007 and 2008 NTP rodent studies of Cr(VI) administered in drinking water, treatment-related effects were reported as histiocytic infiltration and diffuse epithelial hyperplasia in the duodenum of mice and the sole duodenal diagnosis listed for rats was histiocytic infiltration. Based on the present review, there appeared to be 5 distinct effects of Cr(VI) on the intestinal mucosa: villus histiocytic cellular infiltration, villus atrophy/blunting, villus enterocyte vacuolation, villus single-cell necrosis, and crypt epithelial hyperplasia. These 5 diagnoses more comprehensively characterize the morphologic changes that were evident in the examined sections. Importantly, these proposed diagnoses are consistent with the findings described in the texts of the original NTP studies. For example, while atrophy/blunting was not scored directly, the duodenal villi of exposed mice were characterized as “short, broad, blunt …” (NTP 2008b, p. 61). Elsewhere, NTP noted that “epithelial cells lining the tips of the villi of many of the exposed mice were swollen and had vacuolated cytoplasm” (NTP 2007, p. 46). This similarity in observed effects across studies but with different diagnostic reporting highlights the value of peer review for standardizing nomenclature and diagnostic criteria (Morton et al. 2010; Ward et al. 1995). Moreover, such peer review has been “recommended when important risk assessment or business decisions may be based on pathology interpretations in nonclinical studies…and may add value for mechanistic and investigative studies with pathology endpoints …” (Morton et al. 2010, p. 1121). Indeed, these intestinal lesions have been used to inform the derivation of safe drinking water standards for Cr(VI) (U.S. EPA 2010; Thompson et al. 2014; Health Canada 2015).

This review of intestinal lesions in B6C3F1 mice establishes consistency of Cr(VI)-induced non-neoplastic effects across studies. The incidences of treatment-associated non-neoplastic intestinal lesions were similar at comparable milligram per kilogram SDD doses in the NTP (2007) and Thompson et al. (2011) studies (80–89 mg/kg SDD; Table 2). Lesions were slightly greater in male mice than in female mice in the NTP (2007) study, and lesions in female mice were slightly lower in the NTP (2007) study than in female mice from Thompson et al. (2011). Interestingly, villus enterocyte vacuolation and single-cell necrosis were not evident in female NTP mice but were evident in male NTP mice and in female mice from Thompson et al. (2011). In general, atrophy/blunting and crypt hyperplasia occurred concurrently in both studies and in both sexes, whereas villous vacuolation and necrosis occurred primarily at the highest doses. In mice from the 2-year NTP study (Table 4), atrophy/blunting again tended to occur concurrently with crypt hyperplasia. Although not observed in the 13-week NTP study, villus enterocyte vacuolation and single-cell necrosis were observed at the higher doses of both sexes in the 2-year NTP study.

Vacuolation and necrosis in the duodenal villi of mice in the higher dose groups likely implies that the toxic effects of Cr(VI) resulted in the initial death of mucosal epithelial cells (by single-cell necrosis/apoptosis) and increased luminal exfoliation, whereas less overt toxicity occurred at lower Cr(VI) concentrations. Such villus enterocyte loss could lead to crypt epithelial cell proliferation as a compensatory response, perhaps unaccompanied by obvious changes in villus height and structure in the low-dose animals. With increasing Cr(VI) concentrations, accelerated cell loss would eventually exceed the capacity for crypts to regenerate villus enterocytes. Indeed, when intestinal damage is mild, “increased activity of the crypt replication zone compensates for the increased cell loss and there is little detectable change, although the crypt zone increases in length and in mitotic activity…. When cell loss is more severe and more rapid, crypt hyperplasia increases further, enterocytes migrating upwards to cover villi do not have sufficient time to mature and tend to remain crowded together. The villi become shorter and broader …” (I. Brown 2013). Blunting/atrophy is also consistent with a typical response of the small intestine to mucosal epithelial cell loss, which is contraction of the villus in order to maintain barrier function (C. C. Brown, Baker, and Barker 2007; Moore, Carlson, and Madara 1989). Although atrophy/blunting can result from damage to the crypt compartment, this is unlikely the case for Cr(VI) because (1) vacuolation and individual cell necrosis were observed only in the villi of Cr(VI)-treated animals, (2) structural changes in the villi were relatively mild, which is inconsistent with the hallmark severe villus atrophy associated with damage to the proliferative crypt compartment (e.g., ionizing radiation or canine parvoviral enteritis), and (3) crypts of Cr(VI)-treated animals were lined by hyperplastic rather than attenuated epithelium.

Cr(VI) is well known to cause cytotoxicity and genotoxicity *in vitro* (Nickens, Patierno, and Ceryak 2010; Zhitkovich 2005), including in intestinal Caco-2 cells (Thompson, Fedorov, et al. 2012). Thus, it is expected that Cr(VI) could cause toxicity at sites of contact and absorption. Most nutrient and pharmaceutical absorption occurs in the duodenum and proximal jejunum, with far less absorption in the stomach (DeSesso and Jacobson 2001; I. Brown 2013; Mudie, Amidon, and Amidon 2010). Consistent with the location of histopathological lesions in this review, X-ray fluorescence microscopy conducted on intestinal sections of mice from the Thompson et al. (2011) study exposed to 520 mg/L SDD for 13 weeks revealed chromium fluorescence predominantly in duodenal villi, with only near-background levels detected in the crypt region (Thompson, Seiter, et al. 2015). A separate analysis of mice from the Thompson et al. (2011) study, which employed Feulgen staining to assess cytogenetic damage, found no dose-related increases in micronuclei or karyorrhectic nuclei in the crypt but did report significant elevations in micronuclei and karyorrhectic nuclei in villi at ≥60 mg/L SDD (equivalent to ≥20 ppm Cr(VI)^1^; O’Brien et al. 2013). More recently, a second *in vivo* micronucleus assay found no increase in crypt micronuclei in duodenal sections from mice exposed to ≤520 mg/L SDD (Thompson, Wolf, et al. 2015). Taken together, these results further support toxicity limited to the site of contact/absorption.

The review of intestinal lesions in F344 rats further establishes consistency of Cr(VI)-induced non-neoplastic effects across studies. Crypt hyperplasia was present in male rats exposed to ≥32 mg/kg SDD and female rats exposed to 61 mg/kg SDD in the NTP (2007) 13-week study. Atrophy/blunting was not reported originally nor was it evident during this review in the 13-week study. However, atrophy/blunting and crypt hyperplasia were observed in male and female rats of the highest dose groups in the NTP 2-year study (NTP 2008b; Table 5). Additionally, low incidences of vacuolation and individual cell necrosis were present in rats of the highest dose groups. Although it is not possible to be certain if the single cell necrosis observed in such small numbers from the NTP studies is a test article effect, the dose related trend and absence of similar lesions in the control rats supports the view that the lesion is exposure related. As in the case of mice, these findings support that toxicity in rats occurred exclusively in the villi and that villus damage resulted in crypt hyperplasia. Although the incidence of crypt hyperplasia was higher than atrophy/blunting in some groups, this suggests that the magnitude of compensatory cell proliferation equaled or exceeded that of enterocyte loss in such instances.

This current review indicates that Cr(VI) induced qualitatively similar intestinal lesions in both B6C3F1 mice and F344 rats, with effects in the latter appearing much milder than the former. This gradient of effect establishes consistency of non-neoplastic intestinal lesions across species. It is likely that rats did not develop intestinal tumors because the severity of non-neoplastic lesions was milder than in mice, and any threshold necessary for progression of carcinogenesis was not reached in rats. The incidences of crypt epithelial hyperplasia in the samples we examined from NTP 2-year studies at ≥17 mg/kg SDD were 10/11 (91%) and 9/10 (90%) in female and male mice, respectively (Table 4), and 2/15 (13%) and 8/11 (73%) in female and male rats, respectively (Table 5). Although the incidence of hyperplasia in male rats at the highest Cr(VI) drinking water concentration reached 73%, the severity scores of crypt hyperplasia and villus lesions were lower in rats relative to mice (Figure 4). According to Stout et al. (2009), male rats and mice received ∼17 mg/kg SDD at their highest respective Cr(VI) drinking water concentrations, yet non-neoplastic effects in rats were milder than mice (Figure 4). Considering that each stem cell division carries some probability of mutation (Cohen and Ellwein 1990; Tomasetti and Vogelstein 2015), the overall duration of hyperplasia, severity of hyperplasia, and incidence of hyperplasia may increase the risk of tumorigenic response in each test group.

In summary, this review demonstrated that the non-neoplastic histopathologic effects of Cr(VI) in the intestines of mice and rats of the NTP (2007, 2008b) and Thompson et al. (2011), and Thompson, Proctor, et al. (2012) studies were all qualitatively similar, which suggests that the findings were pathogenically interrelated. Specifically, villus atrophy/blunting, enterocyte vacuolation, single-cell necrosis, and crypt epithelial hyperplasia portray a process in which chemically induced villus enterocyte cytotoxicity resulted in regenerative crypt epithelial hyperplasia. This sequela of events, which were more prevalent and severe in mice than rats, could have contributed to the development of duodenal neoplasms in mice.

## Note

1. The average Cr(VI) concentration in U.S. drinking water is 0.001 ppm (Thompson, Seiter, et al. 2015).
